# Passive leg cycling and electrical stimulation cannot preserve strength in sepsis

**DOI:** 10.1186/s13054-018-2226-3

**Published:** 2019-02-08

**Authors:** Pierre-François Laterre, Cheryl Hickmann, Diego Castanares-Zapatero

**Affiliations:** St Luc University Hospital, Université Catholique de Louvain, Avenue Hippocrate 12, 1200 Brussels, Belgium

**Keywords:** Critically ill, Exercise, Electrical stimulation, Muscle, Strength

To the Editor,

In a prospective, randomized, single-center trial including a total of 314 patients, Fossat et al. reported that early in-bed leg cycling exercises together with quadriceps muscles electrical stimulation added to standard rehabilitation program did not improve global muscle strength of critically ill patients at the time of intensive care unit (ICU) discharge [1]. Although the authors adequately pointed out the limitations of their study, the take-home message to critical care personnel may be misleading. Indeed, there are additional aspects in the intervention methods, baseline characteristics, and concomitant therapies that may have modified the observed results and conclusions.

First, passive cycling is unlikely to be able to stretch muscles and therefore no real benefit to muscle strength could be expected. In the study by Burtin et al., exercises using a cycle ergometer were in fact active in up to 87% of the sessions [2]. Second, the duration of this passive cycling was short. In our study, as sedative medication use was restricted, two 30-minute sessions of active/passive cycling were performed by our patients with septic shock and were associated with muscle mass preservation [3]. Third, more than 75% of the patients studied had continuous sedation and 20% had a continuous muscle relaxant infusion suspected to be associated with ICU-acquired weakness. Fourth, electric muscle stimulation has been shown to be ineffective in many critically ill patients, especially because of tissue edema and reduced motor response. In the studied population presented by the authors, more than 60% had sepsis or shock and these conditions are known to be associated with a poor electric response [4, 5]. In addition, the proportion of patients with sepsis was significantly greater in the intervention than in the usual-care group (69% versus 56%, *P* = 0.02). Finally, the passive and active exercises applied in the usual-care group already represent an important intervention, and as pointed by the authors, a quarter of the patients had close to maximal expected muscle strength at discharge and a ceiling effect cannot be excluded.

In our opinion, the conclusion of the study should instead be that short-duration passive leg cycling and electrical quadriceps stimulation did not improve muscle strength in critically ill patients who have sepsis and who need prolonged sedation.

## Abbreviation

ICU: Intensive care unit
